# Supplementary material for: Impact of perceived factors of coronavirus infection on COVID-19 vaccine uptake among healthcare workers in Ghana—Evidence from a cross-sectional analysis
Source: PLoS One. 2025 Feb 12;20(2):e0318662. doi: 10.1371/journal.pone.0318662 (PMC11819584; doi:10.1371/journal.pone.0318662)
Supplement: S1 Table — (DOCX) [file pone.0318662.s001.docx]

***S1. Table.*** *Chi-square test of associations between sociodemographic characteristics and health worker vaccine uptake*

| **Variables** | **P-value** | **X^2^** | **df** |
| --- | --- | --- | --- |
| Age | 0.928 | 0.456^a^ | 3 |
| Sex | 0.780 | 0.078^a^ | 1 |
| Marital status | 0.148 | 5.352^a^ | 3 |
| Educational Status | 0.564 | 2.039^a^ | 3 |
| Residence of respondent | 0.577 | 0.312^a^ | 1 |
| Category of Health worker | 0.001* | 21.962^a^ | 5 |
| Known comorbidity | 0.030* | 4.724^a^ | 1 |
| COVID-19 infection status | 0.310 | 2.341^a^ | 2 |

Keys: *X^2^ = chi square df= degree of freedom, * = statistically significant*
